# The Influence of Applied Voltage on Drying Kinetics, Quality, and Mathematical Modeling of Ginger During Electrohydrodynamic (EHD) Drying

**DOI:** 10.1002/fsn3.72218

**Published:** 2026-08-06

**Authors:** Mingyu Ding, Jingli Lu

**Affiliations:** ^1^ College of Science Inner Mongolia University of Technology Hohhot China

**Keywords:** electrohydrodynamic drying, ginger, mathematical model

## Abstract

This paper systematically evaluated the effects of electrohydrodynamic (EHD) on the drying characteristics, color, texture, moisture state, volatile components, and drying kinetics model of ginger. The results showed that EHD significantly increased the drying rate, rehydration rate, and effective moisture diffusion coefficient (*D*_*eff*_) of ginger. Among all the evaluated parameters, a voltage of 17 kV can produce the highest quality ginger. The drying rate of the 21 kV treatment group was 4.48 times higher than that of the control group (CG). ln[*MR*] showed a highly linear relationship with time (*R* ^2^ > 0.9). Among the ten thin‐layer drying kinetics models, Midilli et al.'s model performed the best. Under 17 kV conditions, the best color retention was achieved for dried ginger. EHD drying significantly improved the texture of ginger. LF‐NMR results showed a significant decrease in free water and an increase in bound water. Compared with the CG group, EHD showed good retention of most terpenoid compounds in dry ginger. SwissADME predicted six volatile compounds with good drug‐like properties. Based on this study, it has been confirmed that the EHD technology has a good application in the ginger industry, providing theoretical support and experimental basis for the further expansion of EHD technology in the field of food drying.

## Graphical abstract

The graphical abstract systematically outlines the experimental design and major findings of this study. On the left is the conventional natural air‐drying method used as the control group; the middle section illustrates the Electrohydrodynamic (EHD) drying process under voltage gradients of 13, 17, and 21 kV, followed by four analytical dimensions: drying kinetics, color assessment, texture analysis, and detection of volatile organic compounds via HS‐SPME‐GC–MS. By visualizing the data results across these four dimensions, statistical analyzes are finally conducted. The study demonstrates that EHD drying accelerates moisture removal and improves product quality, with the 17 kV treatment achieving the best balance in drying rate, color, texture, and flavor retention.

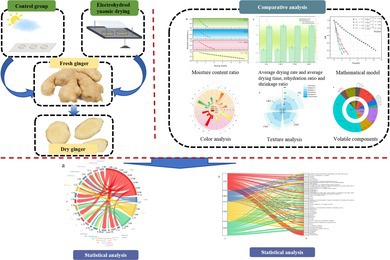

## 1 Introduction

Ginger (*Zingiber officinale Roscoe*), a perennial rhizomatous herb, has a long‐standing history in Asia, particularly in China, India, and Japan, serving as a staple condiment in traditional festivals and folk diets. Its pungent and warm properties not only make it a valued flavoring agent in daily life but also imbue it with symbolic meanings of health, warmth, and vitality, exerting a profound influence on regional culinary cultures and traditional medical systems. Fresh ginger contains a high wet basis (80%–90%), rendering it susceptible to decay, nutrient loss, and quality deterioration during storage. Drying is a critical step in the transportation and deep‐processing of ginger, as it effectively inhibits spoilage, extends shelf life, and thus directly underpins the development of the ginger industry. Mo et al. (2026) compared the effects of hot air drying (HAD) and hot air‐assisted radio frequency drying (HARFD) on the drying performance and mass transfer kinetics of ginger slices. Their findings indicated that under optimized process parameters, HARFD enhanced drying efficiency while preserving superior product quality. Kumar et al. (2025) evaluated solar drying techniques for ginger and turmeric; relative to conventional dryers, solar dryers reduced energy consumption by 75% and lowered carbon dioxide emissions. Chanchula et al. (2025) investigated the pretreatment effects of cold plasma jet (CPJ) and dielectric barrier discharge (DBD) prior to hot air drying of ginger slices. A 15‐s short‐duration pretreatment was found to increase total phenolic and flavonoid contents in dried ginger, accelerate drying rates, and enhance antibacterial activity. These studies have multi‐dimensionally analyzed the impacts of diverse drying technologies on the drying characteristics and quality parameters of ginger, yielding promising experimental outcomes. However, HARFD technology carries risks of quality degradation if parameters are improperly calibrated, and its reliance on radio frequency coupling equipment entails high costs for equipment, maintenance, and process control. Solar drying is highly dependent on meteorological conditions (e.g., solar irradiance, temperature, humidity), making it challenging to stabilize the quality of dried ginger during cloudy periods or seasonal fluctuations. Thus, the development of a novel ginger drying technology that is efficient, high‐quality, and cost‐effective holds significant practical importance and application value for the ginger industry.

Electrohydrodynamic (EHD) drying is a process where a high‐voltage power supply is applied to two electrodes (the emitter electrode and the collector electrode), causing the air around the discharge electrodes to ionize. This is a non‐thermal drying process, and its energy consumption is much lower than that of traditional methods (Shivamurti et al. 2024). The electric field guides the ions from the emitter electrode to the collector electrode (the primary air current). The Coulomb force accelerates the ions, and subsequent collisions with neutral air molecules induce an ion wind. The ion wind disturbs the boundary layer and enhances the convective heat and mass transfer between the drying products (Zafar et al. 2023). In recent years, researchers have introduced EHD into the drying field and conducted a series of explorations, achieving phased results (Zhang, Ding, et al. 2025; Wang and Ding 2023; Xiao and Ding 2022; Lan et al. 2025; Zulhaj et al. 2025; Guan et al. 2024). Bai et al. (2025) used ultrasonic pretreatment coupled with EHD for drying of yam, and the study showed that the EHD technology not only can increase the drying rate of yam, but also has better color retention and more abundant and diverse volatile aroma components. Lian et al. (2025) evaluation results showed that drying at 19 kV resulted in a higher drying rate and better color in dried potato products, and also improved the rehydration rate. However, high voltage can damage cell and protein structures and alter the profile of volatile organic compounds. Zhu et al. (2024) found that voltage can change the volatile component content inside ginger during the EHD drying process, but there is a lack of systematic research on other properties, such as material texture characteristics and internal moisture state. Recent research results on EHD indicate that this technology has significant advantages such as fast drying speed, excellent product quality, and low energy consumption, and is considered a highly promising new non‐thermal drying technology (Dalvi‐Isfahan et al. 2023). However, there are relatively few research reports on the drying characteristics, color changes, texture properties, and volatile components of ginger under the action of EHD. Therefore, whether the EHD drying technology can continue to exert its unique advantages in the ginger drying field still requires further in‐depth research and exploration.

Mathematical models have played a crucial role in the analysis of mass transfer phenomena and in the study of drying kinetics, new design, improvement, and optimization of drying systems. Researchers have proposed mathematical modeling to describe the behavior of dehydrated foods in terms of their drying characteristics. Some semi‐theoretical and empirical models have been developed to describe the EHD drying processes of different products (Dinani et al. 2014). Yawe et al. discovered through experimental research on banana drying that the *R* ^2^ values of the Midilli et al. model were all higher than 0.9, and it could better simulate the banana drying process (John et al. 2025). Veeramanipriya fitted the humidity ratio using nine fitting models and evaluated the goodness of fit of each model, finding that the MHSD model performed the best (Veeramanipriya et al. 2025). Shah, M. conducted a study on the effects of EHD drying with electrode distances of 3 cm, 5 cm, and 7 cm on the microstructure of turmeric slices and the retention of curcumin. The experiments showed that the EHD drying with an electrode distance of 3 cm (at 14 kV) had the best effect, which could maintain the integrity of cell structure to the greatest extent, reduce shrinkage, and retain curcumin (Shah and Srivastava 2025). However, current research on mathematical modeling of fluid dynamics drying is relatively insufficient, and there are no reports of systematic mathematical modeling of the kinetics of ginger drying processes.

This study presents an experimental investigation into the EHD drying of ginger, with the objective of systematically evaluating its influence on key quality attributes, including drying kinetics, color evolution, mechanical properties, volatile compound profile, and internal water distribution. To quantitatively characterize the drying behavior, ten widely adopted thin‐layer drying models, commonly applied in the drying research of medicinal plants, were comparatively assessed. The most appropriate empirical model was identified and validated, thereby establishing a robust predictive framework for ginger drying kinetics and offering novel methodological insights for advancing ginger processing technologies.

## 2 Materials and Methods

### 2.1 Experimental Materials and Methods

The ginger used in the experiment was freshly purchased from a fresh supermarket near Inner Mongolia University of Technology. The initial moisture content of the ginger was determined using a rapid moisture tester, which was 95.00% ± 0.50% wet basis. The fresh ginger was washed and the outer skin was removed, then cut into cylindrical slices with a diameter of 20 mm and a thickness of 2 mm. The pretreated ginger slices were weighed, their initial weight was recorded, and the initial moisture content of the ginger was determined using the rapid moisture tester. Different drying experiments were conducted under different conditions (0 kV (the control group), 13, 17, 21 kV), and the weight of the ginger was measured every 30 min using an electronic balance. The control group had the same drying conditions as the experimental environment of EHD drying except for a voltage of 0 kV. When the wet moisture content in the material is less than 10%, the material can be stored for a long time. Therefore, the drying experiment is stopped when the moisture content on a wet basis inside the material reaches 10%.

### 2.2 Experimental Equipment

The core part of the drying device mainly consists of a high‐voltage power supply (YD (JZ)‐1.5/50 type, 440 mm × 340 mm × 200 mm), a multi‐needle plate‐electrode system (consisting of 2 electrodes: the upper electrode is a multi‐needle electrode with a needle length of 20 mm, and the distance between the needles in both the horizontal and vertical directions is 40 mm; the lower electrode is a stainless steel plate (840 mm × 440 mm), grounded, and the distance between the upper and lower electrodes is 80 mm).

**FIGURE 1:**
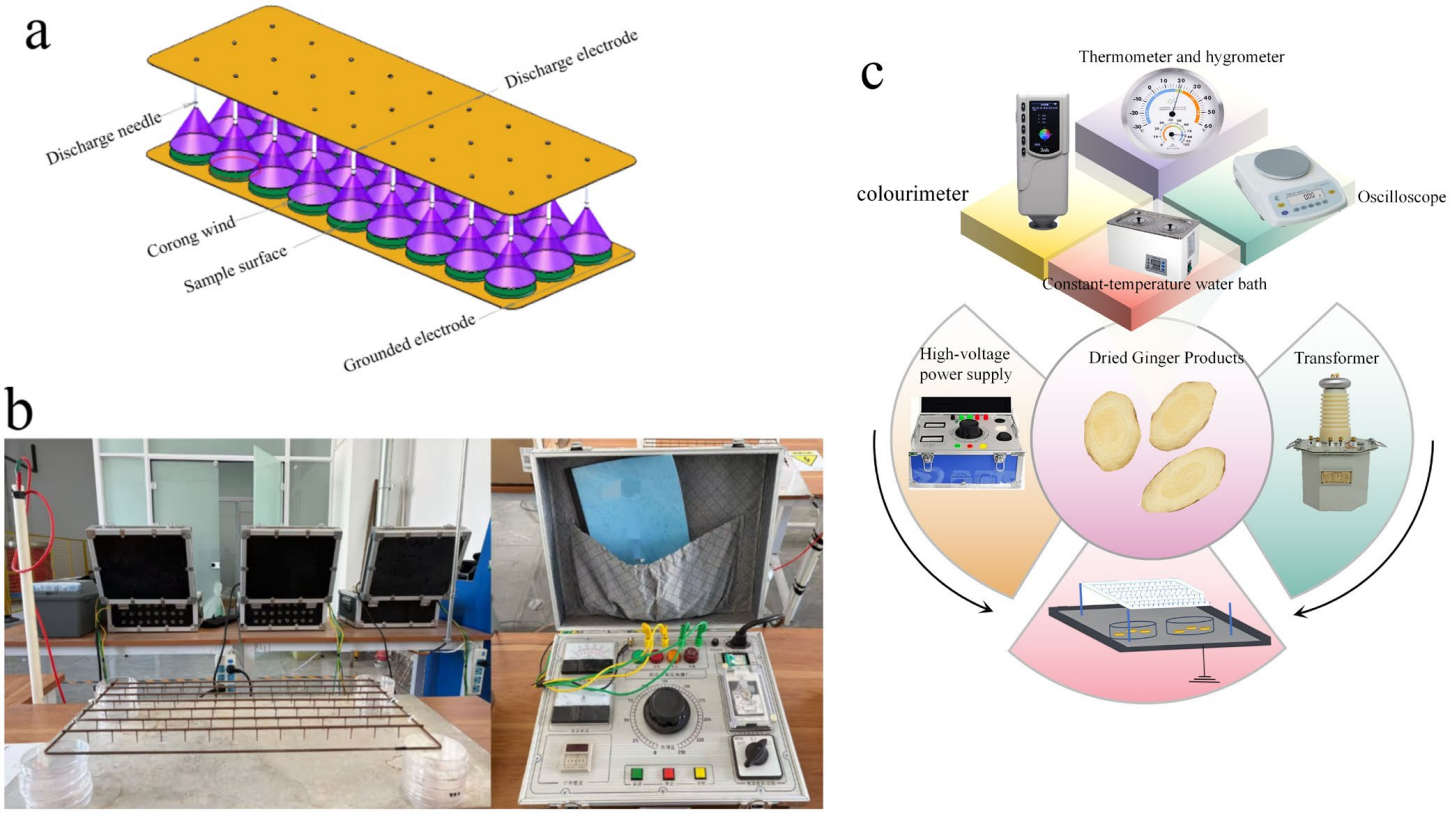
Equipment diagram. (a) Discharge schematic diagram (Dalvand et al. 2014); (b) Physical diagram of equipment placement; (c) Some drying experimental equipment.

Some auxiliary experimental equipment, such as colorimeter (3nh‐ nr60cp, China Shenzhen), electronic balance (BS124S, China Shanghai), constant temperature water bath (DK‐600BS, China Jiangsu), etc., and the equipment schematic diagram and laboratory equipment diagram are shown in Figure 1.

Figure 1a is the schematic diagram of the discharge principle (Dalvand et al. 2014). EHD drying mainly occurs due to the generation of corona wind in the air phase, which leads to the transfer of mass and heat within the dried materials. The corona wind is produced by applying high voltage to small‐sized emitting electrodes, causing the molecules in the nearby air to ionize. These ions are attracted toward the collecting electrode under the influence of the electric field. During their movement, the ions collide with neutral air molecules, resulting in momentum transfer and thus forming the corona wind. The corona airflow impacts the wet materials placed between the two electrodes, thereby achieving drying (Rizki et al. 2026). Figure 1b shows the actual layout of the equipment, and Figure 1c presents the equipment list along with the auxiliary experimental equipment.

During the drying process, the grounded electrode is placed above the drying material, and the electrodynamic drying system can generate discharge phenomena to achieve the drying purpose. The control cabinet can adjust the output voltage of the high‐voltage power supply. In this experiment, the AC voltage was set to 13, 17, and 21 kV, respectively. Since the experiment was conducted under an open indoor condition, the temperature, humidity and wind speed during the experiment were all indoor environmental conditions. The temperature was maintained at 20°C ± 4°C, the relative humidity was at 26% ± 4%, and the natural wind speed was 0 m/s. The drying experiments were conducted in the laboratory, and the natural wind speed is very low, which can ignore the impact on the drying effect of materials. Therefore, the natural wind speed can be approximated as 0.

### 2.3 Drying Characteristics

The dried ginger products after EHD drying were evaluated for the drying process results using mathematical formulas, and the drying characteristics were carefully examined. The results included the changes in ginger sample weight, moisture content, moisture ratio, drying rate, and shrinkage‐rehydration ratio. The overall performance of the drying system was determined based on the changes of these parameters during the experiment. The specific calculation method is shown in Table 1.

**TABLE 1:** Research indicators of drying characteristics.

Formula Name	Equation
Moisture content (Meng et al. 2024)	$m_{g}=m_{0}\left(1-w_{0}\right)$
	$M_{t}=\frac{m_{t}-m_{g}}{m_{g}}$
Moisture ratio (Zhang et al. 2026)	$\mathrm{MR}=\frac{M_{t}-M_{e}}{M_{0}-M_{e}}$
Drying rate (Zhang et al. 2026)	$\mathrm{DR}=\frac{M_{t_{1}}-M_{t_{2}}}{t_{2}-t_{1}}$
Shrinkage ratio (Zhou et al. 2026)	$\mathrm{VSR}\left(\%\right)=\frac{V_{0}-V_{t}}{V_{0}}\times 100$
Rehydration ratio (Bai et al. 2025)	$\mathrm{RR}=m_{1}/m_{2}$

The moisture content and drying rate of the wet base of ginger are referenced from Meng et al. (2024) in their experiment on carrot drying. In the formula, *m*_*g*_ represents the absolute dry weight of ginger (g); *m*_*0*_ is the mass of the material at the initial time (g); *w*_*0*_ is the initial moisture content of the wet base of ginger (%); *M*_*t*_ is the moisture content of the dry base at time *t* (%); *m*_*t*_ is the mass of ginger slices at time *t* (g); *M*_*0*_ is the initial dry base moisture content of the material (g·g^−1^) and *M*_*e*_ is the dry base moisture content of the material when drying reaches equilibrium (g·g^−1^). *M*_*t1*_ is the moisture content of the dry base at time *t1* (g·g^−1^); *M*_*t2*_ is the moisture content of the dry base at time *t*_*2*_ (g·g^−1^).

The shrinkage rate was measured by the quartz sand replacement method to determine the sample volume. The test was conducted three times. In the formula, *V*_*0*_ represents the initial volume of the fresh sample; *V*_*t*_ represents the volume at time t during the drying process.

The rehydration ratio used in the rehydration rate calculation was determined according to the method of Bai et al. (2025) and was slightly modified. In brief, the two pieces of dried ginger were precisely weighed and recorded and placed in an 80 mL beaker and a 37°C water bath. After 3 h, the ginger slices were taken out, the surface water was drained, and the weight was recorded until it no longer changed. In the formula, *m*_*1*_ represents the mass of the ginger after rehydration (g), and *m*_*2*_ represents the mass of the ginger before rehydration (g).

The effective water diffusion coefficient reflects the diffusion ability of water within the material, and is commonly used to predict and analyze the water migration behavior of the material during the drying process. The larger the *D*_*eff*_ value, the faster the water migration speed within the material. By calculating the effective water diffusion coefficient of ginger under different drying conditions, using Fick's second law, the following (Yu et al. 2025) is obtained:

(1) $$\frac{\mathrm{dM}}{\mathrm{dt}}=D_{\mathrm{eff}}\frac{d^{2}M}{dr^{2}}$$

When the drying time is long, *MR* < 0.6. The above formula can be expressed as:

(2) $$\mathrm{MR}=\frac{8}{\pi^{2}}\exp\left(-\frac{\pi^{2}D_{\mathrm{eff}}t}{4L^{2}}\right)$$

In the equation, *D*_*eff*_ represents the effective diffusion coefficient, and *L* is half of the sample thickness. The above equation can be written in logarithmic form as follows:

(3) $$\ln\left[\mathrm{MR}\right]=-\frac{\pi^{2}D_{\mathrm{eff}}}{4L^{2}}t+\ln\left[\frac{8}{\pi^{2}}\right]$$

Based on the relationship between ln[*MR*] and time, we can obtain *D*_*eff*_:

(4) $$k=-\frac{\pi^{2}D_{\mathrm{eff}}}{4L^{2}}$$

The *D*_*eff*_ value is calculated according to Formula (4).

### 2.4 Thin‐Layer Drying Kinetics Modeling

Simulation modeling can help achieve process management and optimization of drying technology. Conducting comprehensive experiments to determine the ideal drying conditions is costly. Therefore, the thin‐layer drying kinetics is selected to express the relationship between water removal and process variables. This kinetics provides an accurate estimation of the drying rate for establishing a drying model. This paper selects ten widely used kinetic mathematical models for food drying (see Table 2) to fit the experimental data of ginger slices in fluid dynamics drying (the relationship between moisture content and time).

**TABLE 2:** Mathematical model.

Model Name	Model equation
Lewis (Newton) (Kusuma et al. 2025)	$\mathrm{MR}=\exp\left(-\mathrm{kt}\right)$
Henderson and Pabis (Kusuma et al. 2025)	$\mathrm{MR}=a\exp\left(-\mathrm{kt}\right)$
Logarithmic (Mang et al. 2026)	$\mathrm{MR}=a\exp\left(-\mathrm{kt}\right)+b$
Parabolic (Polynomial) (Dinani et al. 2014)	$\mathrm{MR}=a+\mathrm{bt}+ct^{2}$
Page (Kusuma et al. 2025)	$\mathrm{MR}=\exp\left(-kt^{n}\right)$
Taghian Dinani et al. (Dinani et al. 2014)	$\mathrm{MR}=a\exp\left[-{\left(\frac{t-b}{c}\right)}^{2}\right]$
Wang and Singh (Mang et al. 2026)	$\mathrm{MR}=1+\mathrm{at}+bt^{2}$
Modified Page (Wang et al. 2026)	$\mathrm{MR}=\exp\left[-{\left(\mathrm{kt}\right)}^{n}\right]$
Midilli et al. (Chappa et al. 2025)	$\mathrm{MR}=a\exp\left(-kt^{n}\right)+\mathrm{bt}$
Weibull (Chappa et al. 2025)	$\mathrm{MR}=\exp\left[-{\left(\frac{t}{b}\right)}^{a}\right]$

The nonlinear regression analysis was carried out by the curve fitting tool in Origin 2024 software. The equation was fitted to the experimental data to determine the equation coefficients and statistical test parameters. Four statistical parameters were used to evaluate the mathematical model. The specific statistical parameters are the reduced chi‐square value ($\chi^{2}$), root mean square error (*ERMS*), squared correlation coefficient (*R* ^2^), and residual sum of squares (*RSS*). The specific calculation formulas are shown in Table 3.

**TABLE 3:** Statistical test parameters.

Statistical parameters	Equation
Reduced chi‐squared (Saraiva et al. 2025)	$\chi^{2}=\frac{\sum_{i=1}^{N}{\left({\mathrm{MR}}_{\exp,i}\;-\;{\mathrm{MR}}_{\mathrm{pre},i}\right)}^{2}}{N-n}$
Root mean square error (Kusuma et al. 2025)	$\text{ERMS}=\sqrt{\frac{1}{N}\sum_{i=1}^{N}{\left({\mathrm{MR}}_{\mathrm{pre},i}-{\mathrm{MR}}_{\exp,i}\right)}^{2}}$
Coefficient of determination squared (Saraiva et al. 2025)	$R^{2}=1-\frac{\sum_{i=1}^{N}{\left({\mathrm{MR}}_{\exp,i}\;-\;{\mathrm{MR}}_{\mathrm{pre},i}\right)}^{2}}{\sum_{i=1}^{N}{\left({\mathrm{MR}}_{\exp,i}\;-\;{\bar{\mathrm{MR}}}_{\mathrm{pre},i}\right)}^{2}}$
Residual sum of squares (Kusuma et al. 2025)	$\mathrm{RSS}=\sum_{i=1}^{n}{\left({\mathrm{MR}}_{\exp,i}-{\mathrm{MR}}_{\mathrm{pre},i}\right)}^{2}$

In the formula, *MR*_*pre,i*_ represents the predicted moisture content of ginger at time *i*; *MR*_*exp,i*_ represents the experimental moisture content of ginger at time *i*; *N* is the number of experimental measurements; *n* is the number of model parameters; ${\bar{\mathrm{MR}}}_{\mathrm{pre},i}$ is the average value of the experimental moisture content.

### 2.5 Color Measurement

Color is a key parameter for evaluating the quality. This study refers to the research conducted by Srivastav et al. (2024) on the color analysis of carrots in the fluid dynamics drying experiments under different electrode distances, and makes some modifications to it in order to measure the color of ginger samples. After calibrating the instrument, five points were taken from the same sample, and the average value was calculated. The color formula is shown in Table 4.

**TABLE 4:** Color determination indicators.

Name	Equation
Color difference (Khatun et al. 2024)	$∆E=\sqrt{{\left(L_{0}-L^{*}\right)}^{2}+{\left(a_{0}-a^{*}\right)}^{2}+{\left(b_{0}-b^{*}\right)}^{2}}$
Chromaticity (Madhankumar et al. 2025)	$C^{*}={\left(a^{*2}+b^{*2}\right)}^{1/2}$
Hue (Wang et al. 2025)	$H^{{}^{\circ}}=\tan^{-1}\left(b^{*}/a^{*}\right)$
Browning Index (He et al. 2026)	$\mathrm{BI}=100\times \frac{\left[\frac{\left(a^{*}+1.75\cdot L^{*}\right)}{\left(5.645\cdot L^{*}+a^{*}-3.012\cdot b^{*}\right)}-0.31\right]}{0.17}$

In the formula, $L_{0}^{*},a_{0}^{*},b_{0}^{*}$ represent the measured values of fresh ginger, while $L^{*},a^{*},b^{*}$ represent the measured values of ginger after drying treatment.

### 2.6 Texture Analysis

Sensory attributes are the key indicators for evaluating the overall quality of dried ginger. Texture, taste, and color are the main parameters for assessment. In recent years, texture measurement has been widely used in the field of food analysis. Su et al. (2025) used a texture analyzer to analyze the texture indicators of cooked beef broth treated with four methods: heat sterilization (PS, HTS) and non‐heat sterilization (HPP, IS), and found that the trends of these parameters for the four sterilization methods were PS maintaining good texture, HTS significantly reducing juiciness and increasing hardness, HPP reducing oxidation but weakening the chewiness, and IS maintaining the chewiness but possibly altering the aroma‐related flavors. Similarly, in Al‐Ali et al.'s study on the preparation of naproxen sodium tablets and powders using different drying techniques, the correlation between the texture characteristics of tablets and their respective powders under different drying methods was explored (Al‐Ali et al. 2020). To meaningfully estimate texture characteristics, researchers and the industry have spent a lot of effort to improve Texture Profile Analysis (TPA) and measurement techniques. TPA tests are based on the imitation of the chewing or chewing process and have a double compression cycle. The typical profile of TPA tests can evaluate the texture characteristics of various fresh and processed foods (Chen and Opara 2013), and determine the following texture parameters: hardness (N/cm^2^), composed of the maximum force required to compress the sample; elasticity (cm), representing the ability of the sample to return to its original state after the force is removed; cohesion corresponding to the degree of deformation that the sample may undergo before breaking (A2/A1, where A1 is the total energy required for the first compression, and A2 is the total energy required for the second compression); viscosity (N) (hardness × cohesion) and chewiness (N/cm), that is, the work required to chew and swallow the sample (Silva Vidal et al. 2025).

This experiment used the TA.XT PlusC (Stable Micro systems England) texture analyzer, with a testing speed of 30 mm/min, a touch force of 3.0 N, and a deformation percentage of 70%. The sample was compressed at a speed of 30 mm/min to a compression ratio of 70%, and the first compression curve (hardness, elasticity, etc.) was recorded; after a set interval of time, the probe returned to the initial triggering position at the same speed; immediately, the second compression (with the same parameters) was performed, and the second compression curve (chewing ability, adhesiveness, etc.) was recorded; all measurements were conducted at room temperature, and each group of tests was repeated three times. After the test, the probe automatically returned to the initial position. The data was saved.

### 2.7 Low‐Field Nuclear Magnetic Resonance (LF‐NMR)

Low‐field nuclear magnetic resonance (LF‐NMR) can be used to characterize the interactions between molecules in food samples, the content of water, the mobility and migration, and can study the different flow states of water in high‐concentration samples and the proton spin–spin (T2) transverse relaxation rate. In recent years, LF‐NMR has made significant progress in evaluating the maturity and damage degree of fruits and foods, the shelf life of food, water migration during food preservation processes, thawing and quick‐freezing processing, and water content testing (Gilbert and Turgeon 2026). Lei et al. conducted non‐invasive root phenotypic analysis based on low‐field nuclear magnetic resonance imaging, detecting the root nodules of rapeseed, which is helpful for the identification of root nodulosis in rapeseed roots (Zhang, Yu, et al. 2025). Zhang et al. (2020) used low‐field nuclear magnetic resonance to determine the internal distribution of water in burdock in hot air and the changes in structure during drying, clarifying the relationship between water loss during burdock root drying and water movement in different states.

Low‐frequency nuclear magnetic resonance (LF‐NMR) has become the preferred method for studying the fluidity of water in food matrices. In this study, the test parameters of LF‐NMR (MesoMR12‐060H, China) were as follows: temperature 30°C, main frequency SF = 12 MHz, 90° pulse time P90 = 7.32 us, 180° pulse time P180 = 11.20 us, offset frequency 01 = 235,731 Hz, signal receiving bandwidth SW = 333.333 kHz, relaxation decay time TW = 1500 ms, cumulative number NS = 16, echo number NECH = 3000, echo time TE = 0.3 ms.

### 2.8 Identification of Volatile Compounds

The volatile organic compounds (VOCs) in dried ginger products were determined by headspace gas–liquid microextraction (SPME) combined with gas–liquid chromatography‐mass spectrometry (HS‐SPME‐GC–MS). The instrument model was GC–MS·7890B‐5977B (Agilent, American); the injection port model was PAL·RTC· Autosampler (CTC·Analytics AG, Switzerland). The determination method was based on the method of Sun et al. (2025) and was adjusted accordingly. The specific protocol was as follows: 3 g of the sample was weighed and placed in a headspace vial. Solid‐phase microextraction was carried out at 50°C for 40 min, followed by desorption at 250°C for 3 min, and then the test was conducted. The chromatographic column was DB‐5MS (Agilent, J&W Scientific 30 m × 0.25 mm × 0.25 um), the injection port temperature was set at 250°C, the carrier gas was helium, the flow rate was 1 mL/min, and the temperature program was as follows: initially maintained at 40°C for 2 min, then increased at a rate of 6°C/min to 200°C, and finally increased at a rate of 15°C/min to 300°C and maintained for 3 min, scanning m/z from 50 to 500.

### 2.9 SwissADME Prediction of ADME Properties of Differentiated Compounds

SwissADME is a tool used to predict the absorption, distribution, metabolism and excretion (ADME) properties of compounds. By submitting the SMILES structure of differentiated compounds, it will automatically standardize and preprocess the structure. Based on physicochemical properties (such as LogP, solubility, permeability), it can predict intestinal absorption and bioavailability, evaluate distribution characteristics such as blood–brain barrier penetration and plasma protein binding, identify metabolic sites and pathways, and assess metabolic stability (Pan et al. 2025). The submission page of SwissADME can be evaluated directly by browsing the web. The required molecular structures can be combined into the main input box. The results are presented in a two‐dimensional chemical structure, standard SMILES and a bioavailability radar that shows the similarity of molecular drugs. The radar considers almost all physical and chemical properties and provides output similar to the given example (Bakchi et al. 2022).

### 2.10 Statistical Analysis

All tests were conducted three times. The experimental data were presented as the mean ± standard deviation (SD). The mean and standard deviation were calculated using SPSS Statistics software, and charts were drawn using Origin 2024 software to evaluate the differences in the drying rate, moisture content, and color data of ginger.

## 3 Results

### 3.1 Drying Characteristics

**FIGURE 2:**
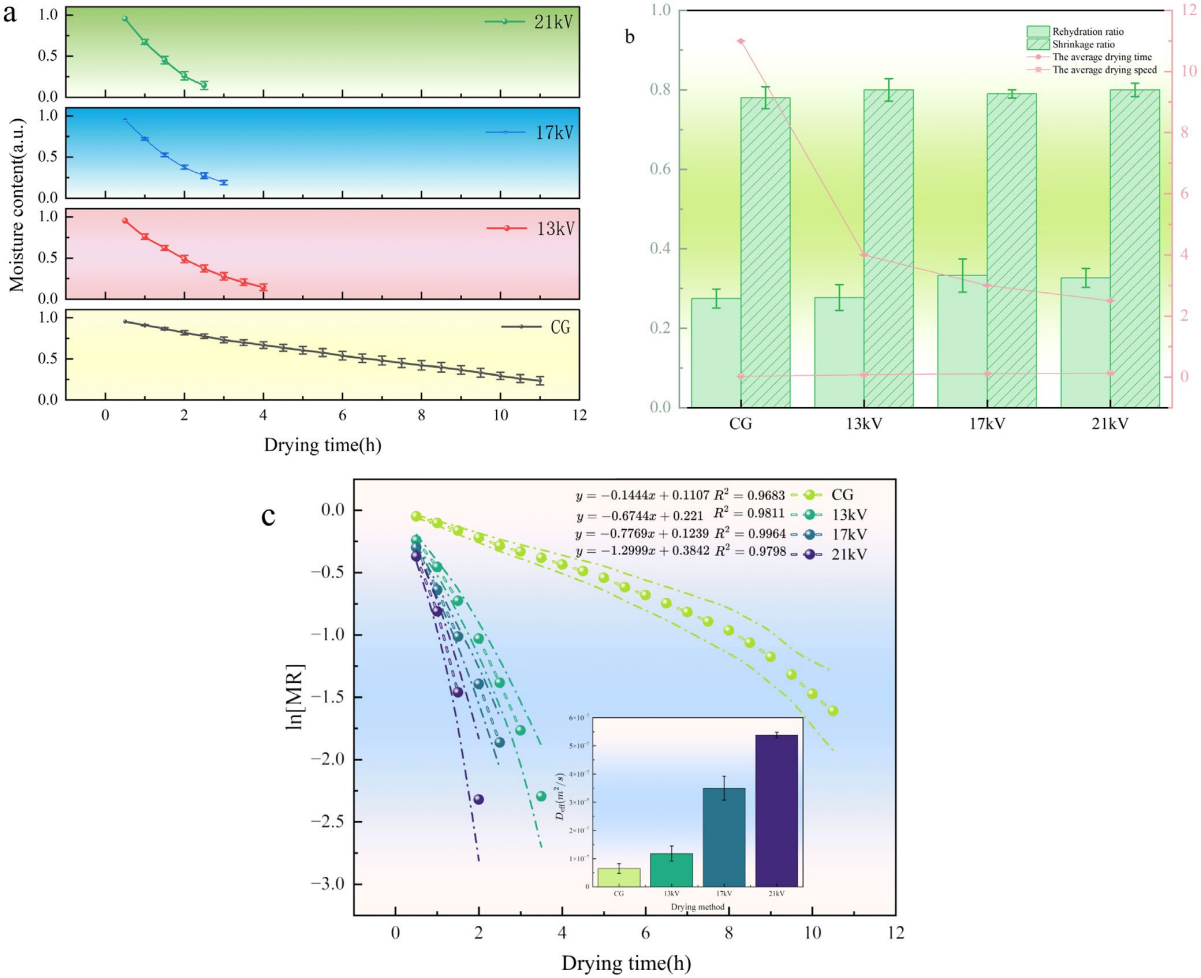
Drying characteristics (a) Moisture content ratio of ginger; (b) Average drying rate and average drying time, rehydration rate and shrinkage rate; (c) Effective moisture diffusion coefficient of ginger.

The moisture content is an important indicator in the drying experiment, as the purpose of drying is to remove the moisture contained in the materials (Kusuma et al. 2023). The curve of the moisture content change of ginger during the experiment is shown in Figure 2a. It can be observed that the change in moisture content of ginger over time has a significant impact (*p* < 0.05). In the early stage of drying, the decrease in moisture content was significant, but the increment gradually decreased as the drying time increased. Compared with the control group, the EHD drying group significantly shortened the drying time of ginger. As the voltage increased, the moisture content gradually decreased, indicating that the voltage gradient has an effect on the drying rate of ginger.

Figure 2b is a composite graph showing the average drying rate, average drying time, rehydration ratio and shrinkage ratio of ginger under different drying methods. The average drying rate of the control group is 0.02900 ± 0.00014 (g·h^−1^), the average drying rate at a drying voltage of 21 kV is 0.1300 ± 0.0015 (g·h^−1^), and the average drying time taken to complete the drying process is 2.5 h. The average drying rate at a voltage of 17 kV is 0.1000 ± 0.0018 (g·h^−1^), and the drying time is 3 h. The average drying rate at a voltage of 13 kV is 0.08000 ± 0.00057 (g·h^−1^), and the average drying time is 4 h. The EHD drying rate is significantly higher than that of CG, and the average drying time is significantly shorter than that of the CG group. The drying speed increases with the increase in voltage, and the average drying time decreases with the increase in voltage. Rehydration ability is an important indicator for evaluating dried products and can also be regarded as an assessment of the damage caused during the drying process. The rehydration ratio is slow or insufficient, which is caused by the decomposition and collapse of the internal tissue (Salehi et al. 2023) From Figure 2b, it can be observed that the rehydration ratio increases with the increase in voltage, with the 17 kV group having the highest value of approximately 0.333, 21 kV approximately 0.327, and CG and 13 kV approximately 0.275–0.278. The rehydration ratio of the EHD group is higher than that of the control group, and the rehydration ratio increases with the increase in voltage. It reaches the highest value at 17 kV, indicating that the 17 kV group causes the least damage to ginger. The shrinkage ratio is concentrated in the range of 0.78–0.80, and there is very little difference among the four groups, with each group's difference not exceeding 0.1, indicating that the change in drying method has a small impact on the shrinkage ratio of ginger.

The mass transfer mechanism during the drying process of food and biological products is usually represented by the effective moisture diffusion coefficient (*D*_*eff*_), which is a key parameter in designing and modeling the drying process. It mainly includes molecular diffusion and vapor pressure‐driven migration. Quantitative analysis of ln[*MR*] revealed a linear relationship between ln[*MR*] and *t*, and a linear simulation equation was established as shown in Figure 2c. The fitting slopes *k* of ln[*MR*] and t obtained from different treatment conditions (CG, 13, 17, 21 kV) within 0–12 h were calculated. In the fitting of ln[*MR*] against time in Figure 2c, the slopes *k* of the four linear simulation equations were −0.1444, −0.6744, −0.7769, and −1.2999, respectively, and the corresponding fitting determination coefficients *R* ^2^ were 0.9683, 0.9811, 0.9964, and 0.9798. All *k* values were negative and the fitting was good, indicating that this model could well describe the relationship between ln[*MR*] and *t*. It was observed that the absolute value of the slope increased simultaneously with the increase in voltage, and the decay rate increased. The *D*_*eff*_ of ginger was obtained based on the relationship between ln[*MR*] and time. The average *D*_*eff*_ values were approximately 6.5 × 10^−8^ m^2^/s for CG, 1.18 × 10^−7^ m^2^/s for 13 kV, 3.5 × 10^−7^ m^2^/s for 17 kV, and 5.38 × 10^−7^ m^2^/s for 21 kV, showing a monotonically increasing trend. The increase in voltage increased the decay rate of ln[*MR*], improved the drying rate, and also increased the *D*_*eff*_, with a larger *D*_*eff*_ indicating that water was more easily diffused from the interior of the sample to the surface. Higher voltage gradients promoted water transfer.

### 3.2 Analysis of Thin‐Layer Drying Kinetics Model

**FIGURE 3:**
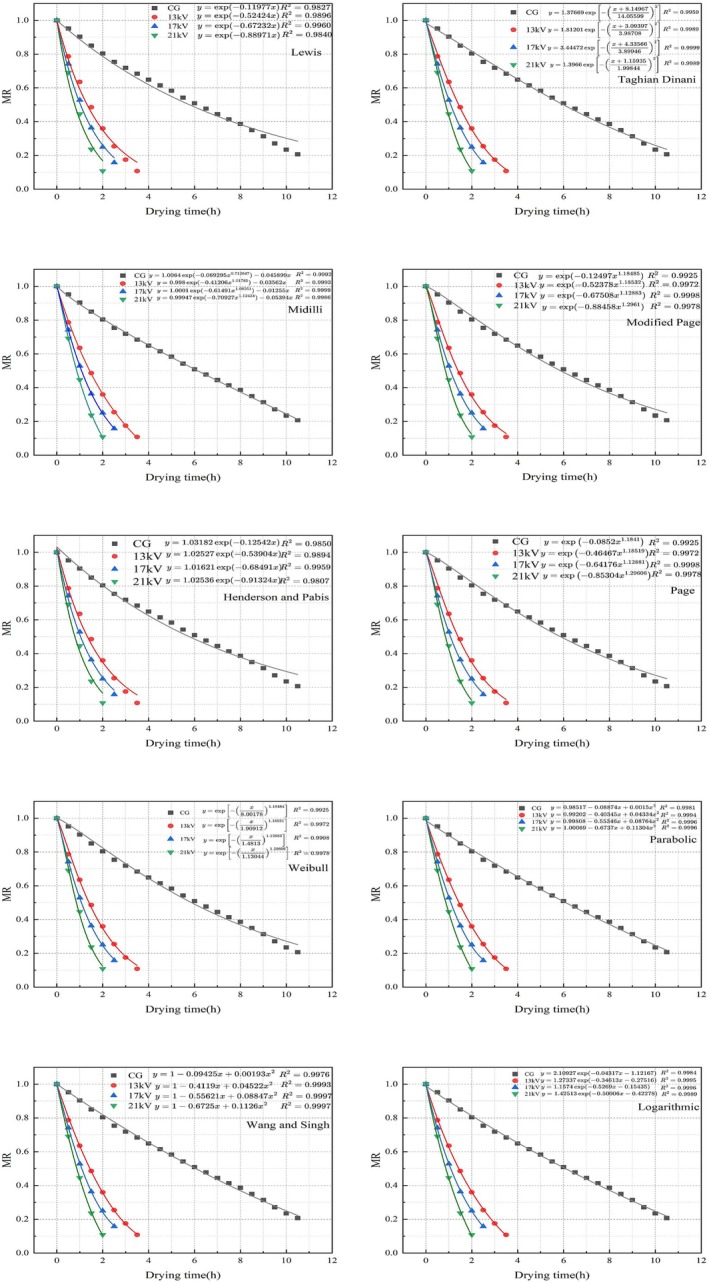
Model fitting curve.

Figure 3 shows the fitting curve of the model, and Table 5 presents the statistical parameters of the model. The Lewis model is too simple, and the fitting curve significantly deviates from the data points. It has a large prediction error for the initial descent speed under high voltage conditions and is not suitable for complex drying processes. However, it performs well for materials with high initial drying and high humidity (Nisha and Ashish Mohite 2025). Henderson and Pabis are more stable than Lewis in most data, but they still cannot fit the tail deceleration stage well, and their efficiency is low in the final stage of the process. The Logarithmic model introduces a constant term *b*, which can handle the tail of the data points but has unstable parameter values. In drying experiments, this model is often used, and it has accurately predicted the drying behavior of basil leaves (Luthra and Sadaka 2021). The Parabolic polynomial form is flexible, but its physical meaning is not as intuitive as the attenuation model. The fitting degree depends on the original data distribution, and its flexibility is poor. Page and Modified Page contain nonlinear combinations of exponents and powers, which can well describe the acceleration or deceleration stages of the drying process. They are very commonly used in food drying modeling and often outperform simple exponential models. The Wang and Singh quadratic polynomial has a high dependence on time, and strict constraints need to be imposed on *MR*. This model is effectively used to describe the drying behavior of parsley leaves and bamboo shoot slices (Singhal et al. 2022). The Midilli et al. and Weibull models show the most stable and closest‐to‐real‐observation fitting. They can describe both the rapid degradation in the early stage and the slow descent in the middle and later stages. From the statistical parameters, the Parabolic, Page, Taghian Dinani et al., Modified Page, Midilli et al., and Weibull models have very excellent fitting effects on the drying experimental data of ginger under high voltage electric field. Most of their *R* ^2^ values are close to 1, while the $\chi^{2}$, *ERMS*, and *RSS* values are very small. Page and Modified Page can also achieve good fitting, but the model is too simple and cannot predict complex experimental situations, making it suitable as a rapid prediction model. Overall, the higher the voltage, the faster the overall drying rate. The coefficients *k* of time *t* in the fitting parameters are all negative, and the absolute values of *k* increase with the increase of the voltage gradient, indicating that the rate of water evaporation or migration per unit time increases. At all voltage levels, the Midilli et al. model performs the most stably and has the optimal statistical parameters. The average *R* ^2^ of the Midilli et al. model is close to 0.999, with the smallest $\chi^{2}$, *ERMS*, and *RSS* values of 5.50 × 10^−5^, 0.00671, and 9.9 × 10^−4^, respectively. The parameter variation range within each voltage group has a small degree of dispersion, enabling it to adapt to more complex experimental situations. Anand et al. found in their study on the kinetics model of potato drying that the drying kinetics fitting effect of the Midilli et al. model was the best, which was the same as the results of this experimental study (Kushwah 2025).

**TABLE 5:** Model statistical parameters.

Model	Group	$\chi^{2}$	ERMS	*R* ^2^	RSS
Lewis	CG	0.2548	0.50475	0.9827	2.06 × 10^−2^
	13 kV	0.0353	0.03131	0.9896	7.17 × 10^−3^
	17 kV	0.0148	0.02004	0.9960	2.01 × 10^−3^
	21 kV	0.0013	0.05975	0.9721	1.4 × 10^−2^
Henderson and Pabis	CG	0.2675	0.02906	0.9850	1.69 × 10^−2^
	13 kV	0.0459	0.03223	0.9894	6.23 × 10^−3^
	17 kV	0.0207	0.04401	0.9959	1.67 × 10^−3^
	21 kV	0.0048	0.04972	0.9807	7.42 × 10^−3^
Logarithmic	CG	0.3739	0.00994	0.9983	1.88 × 10^−3^
	13 kV	1.1310	0.00712	0.9995	2.54 × 10^−4^
	17 kV	1.6092	0.00628	0.9996	1.18 × 10^−4^
	21 kV	2.3572	0.01185	0.9989	2.81 × 10^−4^
Parabolic	CG	1.10 × 10^−4^	0.01046	0.9981	2.08 × 10^−3^
	13 kV	5.37 × 10^−5^	0.00733	0.9999	2.68 × 10^−4^
	17 kV	3.95 × 10^−5^	0.00629	0.9996	1.19 × 10^−4^
	21 kV	5.54 × 10^−5^	0.00745	0.9996	1.11 × 10^−4^
Page	CG	4.21 × 10^−4^	0.02052	0.9925	8.42 × 10^−3^
	13 kV	2.78 × 10^−4^	0.01668	0.9972	1.67 × 10^−3^
	17 kV	2.17 × 10^−5^	0.00466	0.9998	8.69 × 10^−5^
	21 kV	2.88 × 10^−4^	0.01697	0.9978	8.64 × 10^−4^
Taghian Dinani et al.	CG	2.32 × 10^−4^	0.01523	0.9959	4.41 × 10^−3^
	13 kV	1.05 × 10^−4^	0.01027	0.9989	5.27 × 10^−4^
	17 kV	1.39 × 10^−5^	0.00373	0.9999	4.18 × 10^−5^
	21 kV	1.41 × 10^−4^	0.01187	0.9989	2.82 × 10^−4^
Wang and Singh	CG	1.36 × 10^−4^	0.01167	0.9976	2.72 × 10^−3^
	13 kV	6.54 × 10^−5^	0.00809	0.9993	3.92 × 10^−4^
	17 kV	3.08 × 10^−5^	0.00555	0.9997	1.23 × 10^−4^
	21 kV	3.71 × 10^−5^	0.00609	0.9997	1.11 × 10^−4^
Modified Page	CG	4.21 × 10^−4^	0.02052	0.9925	8.42 × 10^−3^
	13 kV	2.78 × 10^−4^	0.01668	0.9972	1.67 × 10^−3^
	17 kV	2.17 × 10^−5^	0.00466	0.9998	8.69 × 10^−5^
	21 kV	2.88 × 10^−4^	0.01697	0.9978	8.64 × 10^−4^
Midilli et al.	CG	5.50 × 10^−5^	0.00671	0.9992	9.9 × 10^−4^
	13 kV	7.06 × 10^−5^	0.00840	0.9993	2.82 × 10^−4^
	17 kV	1.26 × 10^−5^	0.00355	0.9999	2.52 × 10^−5^
	21 kV	1.77 × 10^−4^	0.01329	0.9986	1.77 × 10^−4^
Weibull	CG	4.21 × 10^−4^	0.02052	0.9925	8.42 × 10^−3^
	13 kV	2.78 × 10^−4^	0.01668	0.9972	1.67 × 10^−3^
	17 kV	2.17 × 10^−5^	0.00466	0.9998	8.69 × 10^−5^
	21 kV	2.88 × 10^−4^	0.01697	0.9978	8.64 × 10^−4^

### 3.3 Color Analysis

**FIGURE 4:**
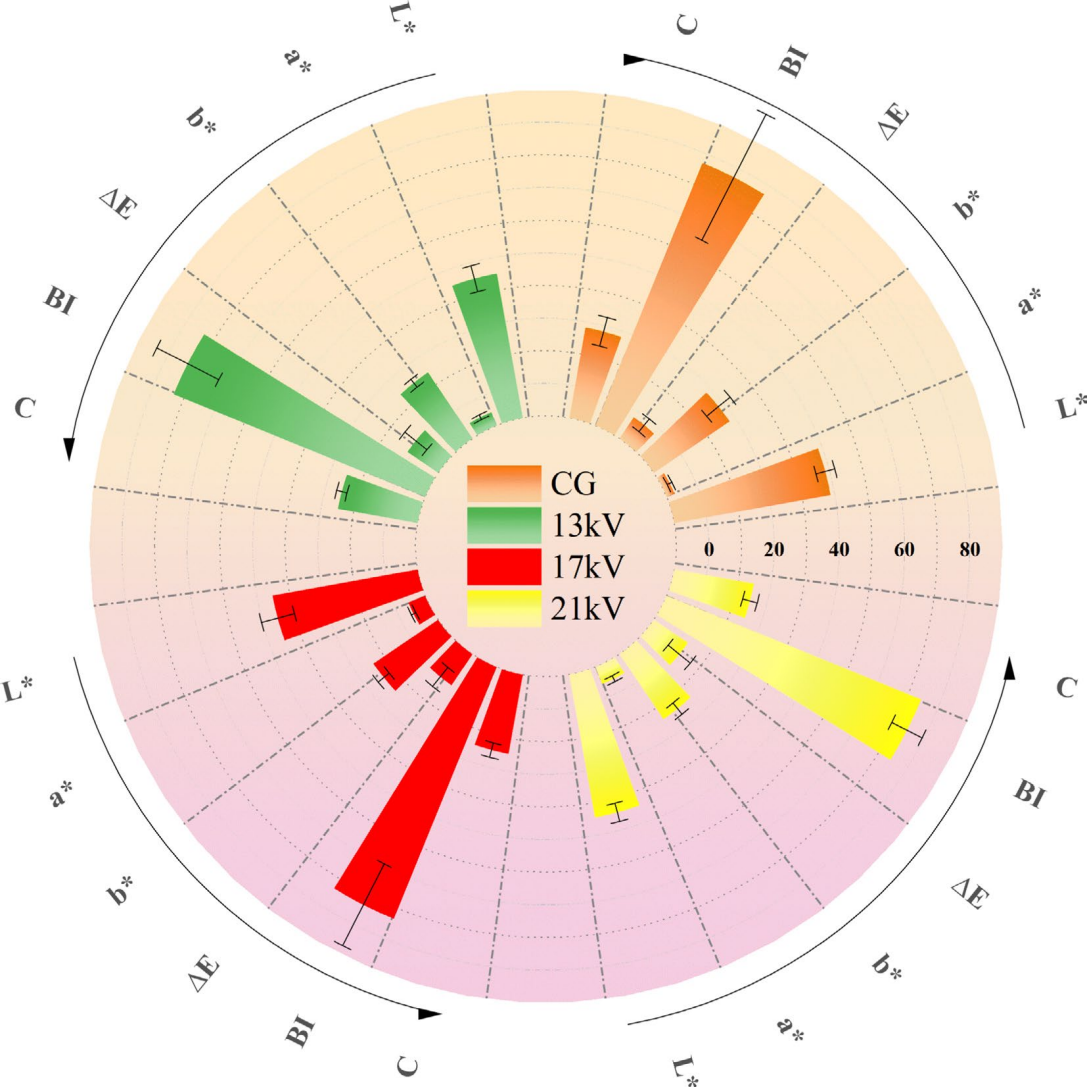
The color parameters of ginger, including L*, a*, b*, *C*, ∆E, and *BI* values.

High‐quality dried ginger products usually maintain high brightness (L*), high yellowness (b*), and low redness (a*). Color, as a fundamental sensory attribute, is directly related to consumers' purchasing decisions and value assessment. Color parameters (L*, a*, b*) show significant differences (*p* < 0.05) in different drying techniques. Chroma (*C*) indicates color intensity, with higher values resulting in more saturated and vivid colors. Hue angle (*H* ^°^) is a key measure of food color, with a value of 0 or 360 representing red, while angles of 90, 180, and 270 correspond to yellow, green, and blue, respectively (Shahimoridi et al. 2025). The color parameters are shown in Figure 4. The 17 kV sample exhibited the best color retention, with the highest L* (48.71), b* (28.07), and a* (2.56), the highest *C* (36.01), and an excellent hue angle *H* ^°^ (86.63), which was closest to the ideal color of high‐quality ginger. The color parameters of each EHD group showed gradually increasing color retention as the voltage increased. In the food industry, the browning of fresh products is a major challenge and is the main factor causing quality decline during post‐harvest processing. This browning process is mainly driven by enzymatic reactions. The main reasons for color deterioration include intense browning processes, such as the Maillard reaction, oxidation, and changes in surface morphology. There was no significant difference in browning rate between the control group and the EHD method (*p* > 0.05), highlighting the key role of the duration of temperature rather than the absolute temperature in color degradation. As the brightness decreased, the color changed from light yellow to brown yellow due to the alkaline decomposition of curcumin into vanillin acid and vanillyl methane (Jeevarathinam et al. 2021).

### 3.4 Texture Characteristics

**FIGURE 5:**
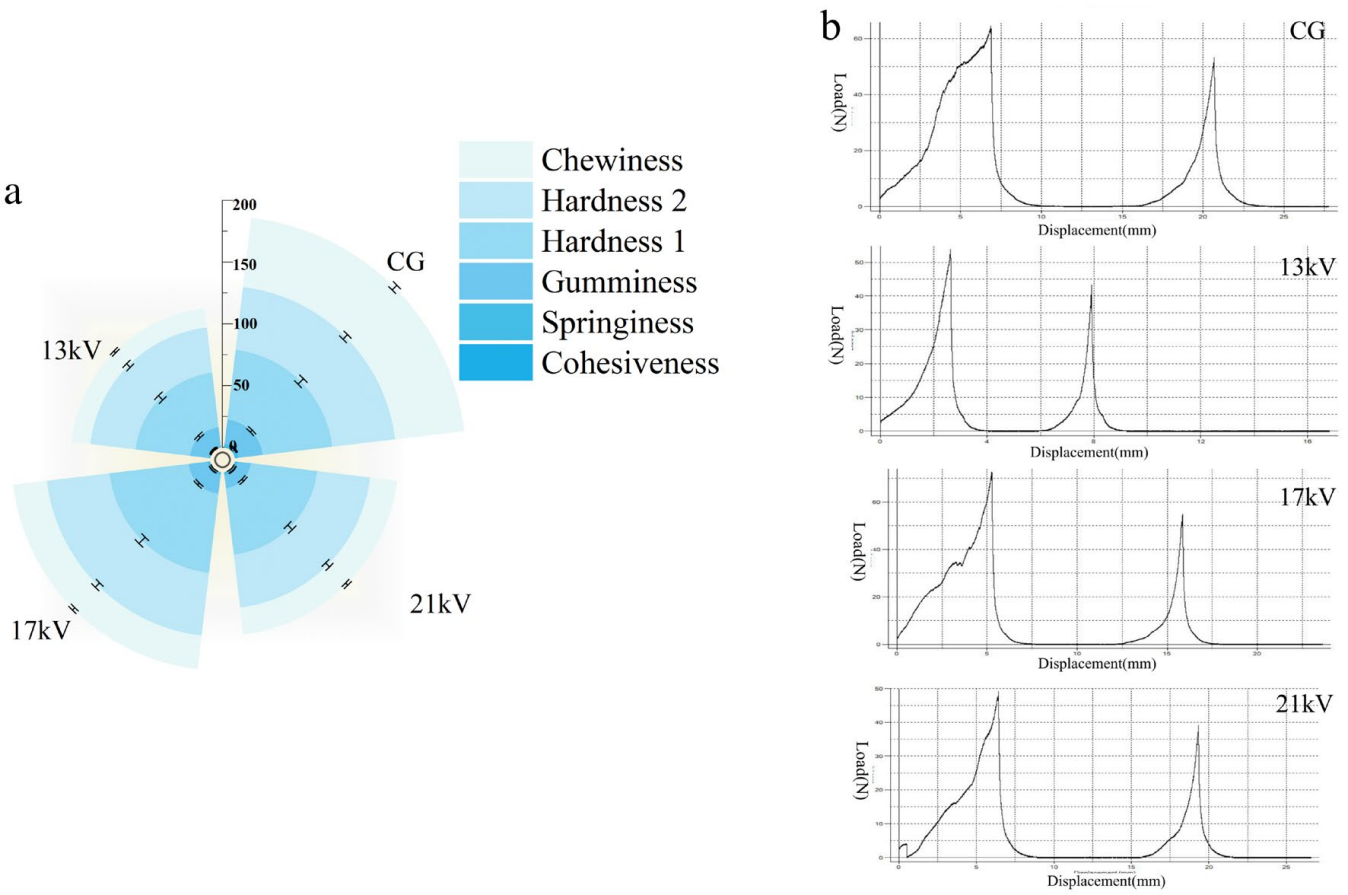
Textural characteristics of ginger under different drying methods (a) Cohesiveness, springiness, gumminess, hardness 1, hardness 2, chewiness values; (b) Stress–strain curves of ginger.

Among the parameters of TPA, key attributes such as hardness, adhesion, elasticity, stickiness and chewability provide reliable methods for evaluating the quality characteristics of dried products, especially in overcoming the consumption barriers faced by patients with chewing difficulties (Zhou et al. 2025). The specific quality characteristic parameters are shown in Figure 5a. It can be observed that compared with the control group, the hardness 1 and hardness 2 of the 17 kV and 21 kV treatment groups have significantly increased. Hardness is a key quality indicator of dried ginger. Adequate hardness can ensure low moisture content, prevent deterioration and achieve the stability of ginger, while minimizing damage during the processing (Mo et al. 2026). The value of the chewability CG group is (56.4 ± 2.7) N. Chewing degree is the amount of energy required during swallowing or chewing. An excessively high chewability will affect the taste and quality of ginger, and the EHD group has a better overall performance in the chewability index. The gap in ginger adhesion between EHD and CG is small, indicating that the drying method has little effect on adhesion. CG has excessively high elasticity and viscosity. In summary, EHD has a better retention effect on the texture of ginger. The 17 kV group performed better, indicating that the appropriate voltage parameters have a significant impact on the quality of ginger dry products.

Figure 5b shows the stress diagrams of ginger under different treatment groups in the texture analyzer. All four curves have two distinct peaks, indicating that ginger undergoes layered failure under the action of the texture analyzer. The damage to the cell membrane can reduce the expansion pressure and thereby lower the hardness (Wang et al. 2022). From the figure, it can be seen that the curve characteristics of the 21 kV group are between CG and 17 kV, and the change in peak distribution is not as significant as that of 17 kV. Overall, the EHD group shows a significantly and consistently changing trend compared to CG in terms of the information observed in the stress diagram, such as peak load, peak displacement, and curve area. Under the 17 kV condition, the two peaks of the curve are clearer, the peak distribution is more stable, and a more uniform pore structure and water distribution may be produced during the drying process.

### 3.5 Moisture State

**FIGURE 6:**
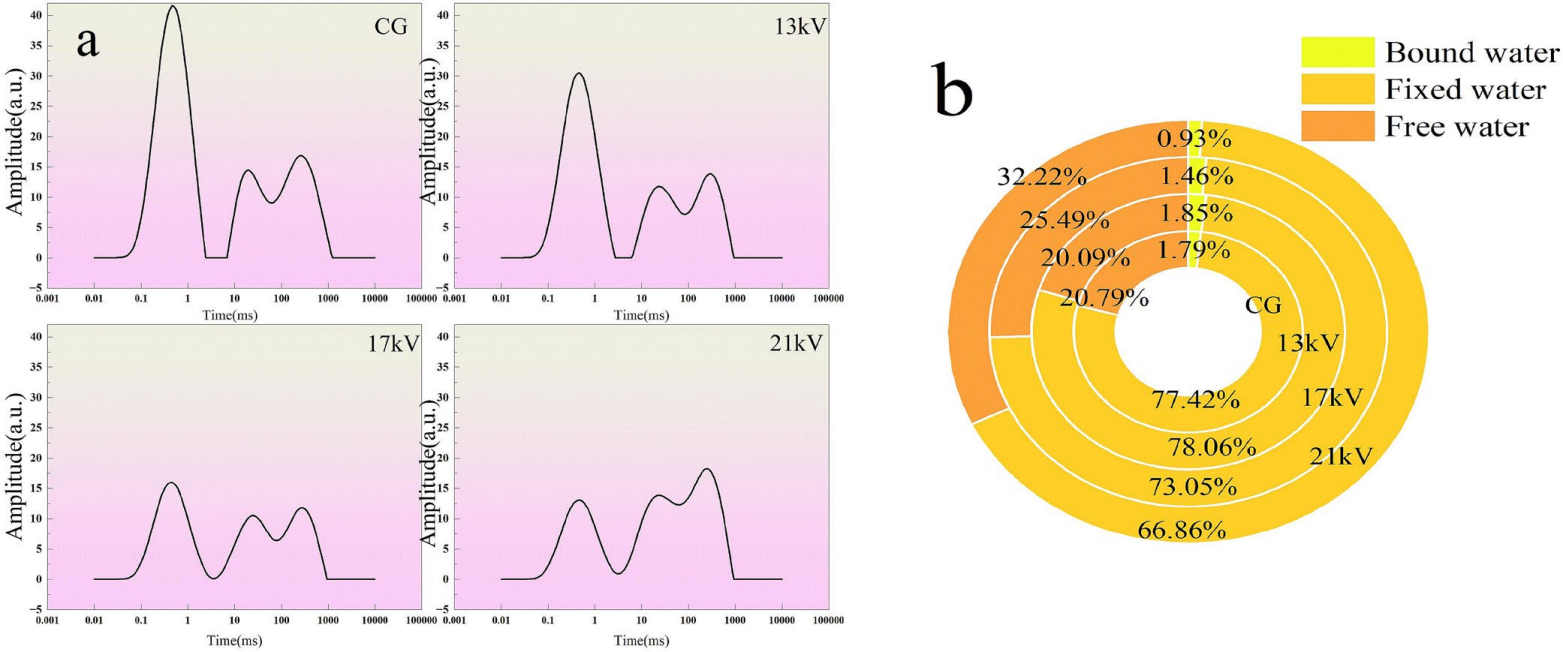
Low‐field nuclear magnetic resonance: (a) *T*_2_ spectrum; (b) Relative water content.

In LF‐NMR, the three forms of water can be classified based on relaxation time: (0.01–1) ms represents bound water, (1–100) ms represents fixed water, and (100–10,000) ms represents free water. The low‐field nuclear magnetic resonance spectrum is shown in Figure 6a. The horizontal axis of the figure represents T2 (milliseconds), and the vertical axis represents frequency. It can be seen that the control group shows more peaks of long T2 (free water), indicating that the sample contains more free water. As the drying voltage intensity increases (13, 17, 21 kV), the peak of free water gradually decreases, indicating that the content of free water decreases. On the other hand, the distribution of bound water (short T2) and hydrated water (medium T2) gradually strengthens, indicating that the moisture gradually transforms into a bound state. Compared to the control group, the signal amplitude in the short to medium relaxation time interval of 13 kV is effectively retained, while the free water signal in the long relaxation time interval is significantly suppressed. At T2≈500 ms, the signal drops from 12.65 a.u. of CG to 10.20 a.u., with an inhibition amplitude of approximately 19.4%; 17 kV further strengthened this trend, and the weakly bound water signal in the medium relaxation time interval became more concentrated, with the average signal in this interval being approximately 9.2% higher than that of CG, indicating that the binding state of moisture in the material becomes more stable; the peak of the relaxation spectrum at 21 kV shifted more significantly toward the short relaxation time direction. The peak position of the relaxation spectrum shifted from approximately 40.71 ms of CG to 13.10 ms, and the free water signal in the T2 > 1000 ms interval was almost completely suppressed, indicating that at this voltage, drying can remove the free moisture to the greatest extent and stabilize the moisture in the bound state, reducing the risk of subsequent moisture absorption or re‐distribution.

Figure 6b shows the relative moisture content of each treatment group. The pie chart presents the proportions of the three types of moisture as follows: for the CG group, the combined water accounts for 77.42%, the fixed water 20.79%, and the free water 0.93%; for 13 kV, it is 78.06%, 20.09%, and 1.46%; for 17 kV, it is 73.05%, 25.49%, and 1.85%; and for 21 kV, it is 66.86%, 32.22%, and 1.79%. From the trend, the relative proportion of combined water decreases significantly with the increase in voltage (77.42% → 66.86%), while the relative proportion of fixed water increases significantly (20.79% → 32.22%), and the proportion of free water, although always small, shows a slight upward trend (0.93% → 1.79%) among the four points. It can be observed that the typical signal peak of free water (i.e., 100–10,000 ms) decreases with the extension of drying time and shifts to a lower T2 range during the drying process. This indicates that during the heating process, the free water in the ginger slices is removed, and the fixed water and combined water begin to gradually dry (Zeng et al. 2022).

Compared with CG, the relaxation spectrum of EHD has a narrower time distribution range and a more concentrated amplitude peak. After traditional drying, the free water signal with long relaxation time still has some residual, which means that there is still unremoved free water in the material, which is prone to cause material spoilage and quality deterioration (Mo et al. 2026). However, after EHD treatment, the water mainly exists in a bound state, not only prolonging the storage period of the material, but also better preserving the structural integrity and functional characteristics of the material. From the perspective of the efficiency of water migration during drying, EHD accelerates the migration process of water from the interior to the surface through the action of high‐voltage electric field. The relaxation signal after drying completes the transformation from long to short relaxation in a short period of time, indicating that the water migration rate is fast, the drying cycle is short, and the water removal is more thorough.

### 3.6 Volatile Components

**FIGURE 7:**
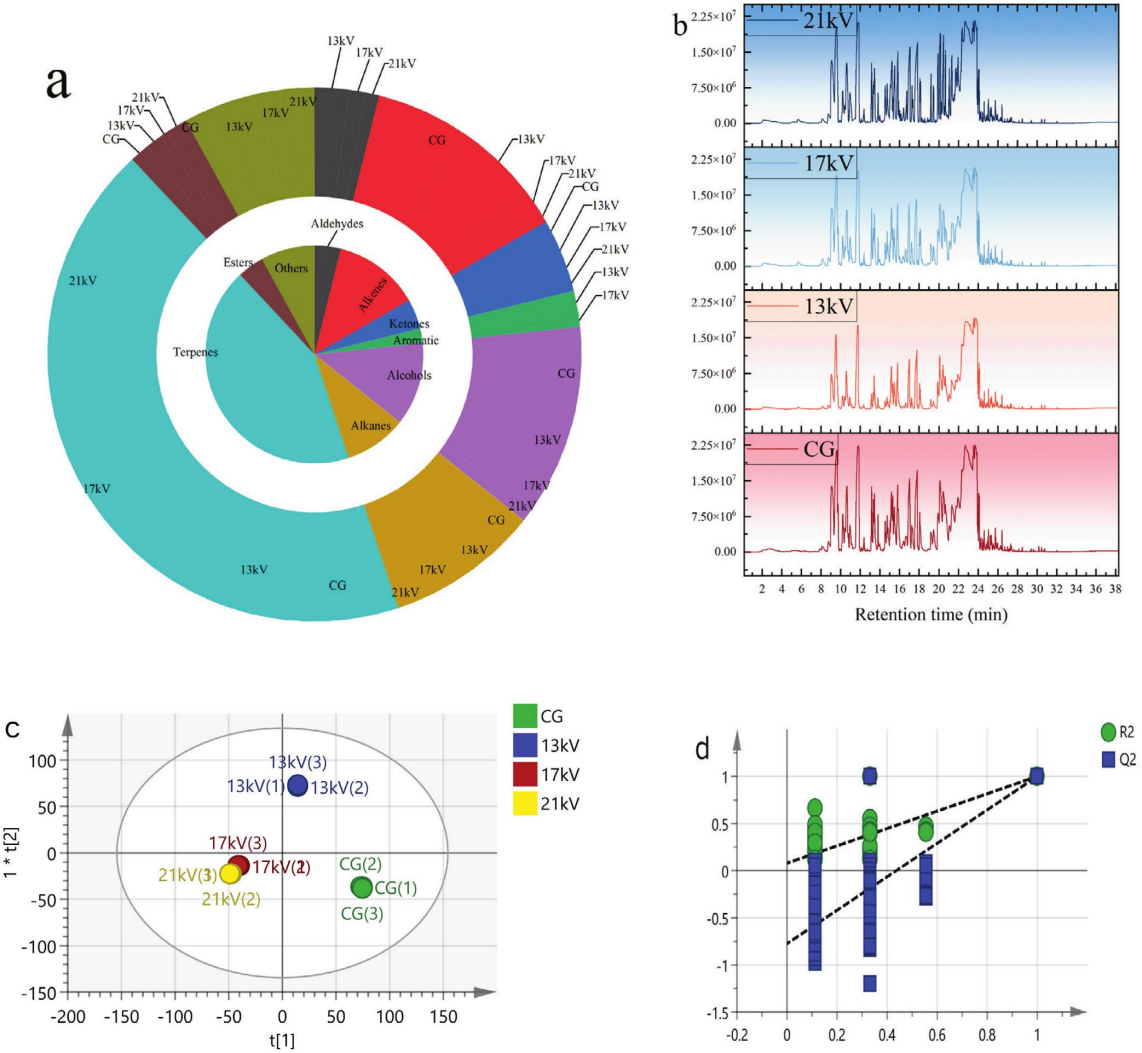
Volatile compound types. (a) Comparison chart of the types of volatile compounds; (b) TIC fingerprint spectrum; (c) OPLS‐DA; (d) Model cross‐validation results.

The VOC in the dried ginger samples of the EHD and CG groups were compared and analyzed using HS‐SPME‐GC–MS, as shown in Figure 7a. It can be observed that terpenoids are the main components of the ginger VOCs. Among them, 46 differential characteristic markers (including 33 terpenes, 2 alcohols, 2 esters, 3 hydrocarbons, 4 aldehydes, and 2 ketones) were selected based on the criterion that the importance of the predictive variables (VIP) values is greater than 1, and detailed analysis was conducted.

Among the four aldehyde volatile substances, Hexanal has a relatively high content. It is a colorless transparent liquid with a strong grassy smell or the characteristic irritating odor of fatty aldehydes. It is a colorless to pale yellow liquid with a strong lemon or citronella‐like characteristic aroma. Citronellal is mainly extracted from natural plant essential oils such as citronella oil (with a content of approximately 30%–40%), lemon grass oil, and eucalyptus leaf oil, and is one of the core contributing components of the aroma of these essential oils. There are the most types of aldehydes in the 13 kV group, and aldehyde compounds were not detected in the control group. EHD can well retain aldehyde compounds.

The content and types of terpenes are the most abundant. Terpenes occupy a central position in the volatile substances of ginger and stand out in terms of variety and abundance. They consist of monoterpenes and sesquiterpenes. A total of 33 terpenic volatile substances were identified under different drying methods, among which the five with the highest content were Camphene, β‐Mychrene, 1,3‐Cyclohexadiene, 5‐(1,5‐dimethyl‐4‐hexenyl)‐2‐methyl‐, [S‐(R*,S*)]‐, [S‐(R*,S*)]‐, (8R,8aS)‐8,8a‐Dimethyl‐2‐(propan‐2‐ylidene)‐1,2,3,7,8,8a‐hexahydronaphthalene, and α‐Phellandreneα‐. Among them, Camphene, β‐Mychrene, and α‐Phellandreneα‐ are monoterpenes, with low boiling points and strong volatility, thereby giving ginger a refreshing aroma (Pang et al. 2017). The quantities of terpenic compounds in ginger samples dried by EHD were mostly higher than those in the CG group, indicating that EHD has a better retention effect on most terpenic volatile substances.

Two types of alcohol compounds were detected. Among them, 2‐Undecanol and Isoborneol had the highest content in ginger VOC. 6‐Octen‐1‐ol, 3,7‐dimethyl‐, acetate was an ester volatile substance with relatively high content in ginger under different drying methods. It is a colorless to pale yellow transparent liquid at room temperature, with fresh lemon, rose or citrus‐like fragrances. It is a common component of daily‐use fragrances. Moreover, it is a permitted edible fragrance allowed by regulations and is mainly used for preparing fruit‐type fragrances such as pears, lemons, peaches, apples, raspberries, and citrus fruits. This compound was only detected in the EHD group samples.

The alkane with the highest content in the experiment, Tricyclo[2.2.1.0(2,6)]heptane, 1,7,7‐trimethyl‐, is an important organic synthetic intermediate that can be used to synthesize a variety of bioactive compounds and has certain application prospects in the fields of medicine and pesticides. The alkene, Cyclohexane, 1‐ethenyl‐1‐methyl‐2,4‐bis(1‐methylethenyl)‐, [1S‐(1.alpha.,2.beta.,4.beta.)]‐, is a raw material for fragrance formulation and can also be used in organic synthesis, serving as an intermediate for the preparation of other complex organic compounds.

The samples were analyzed using HS‐SPME‐GC–MS, and the analysis results are shown in Figure 7b. Figure 7b established a fingerprint spectrum based on the retention time and peak area parameters of volatile organic compounds, depicting the typical TIC of ginger under different drying methods. Most of the adsorbed compounds in HS‐SPME‐GC–MS are distributed within the first 30 min of the TIC. They mainly concentrate in the 10–30 min range. The peaks of TIC in different regions are slightly different. OPLS‐DA can distinguish the sample groups based on the chemical properties of the samples and identify specific compounds that cause the differences between each group (Zhang, Yan, et al. 2025). As observed in Figure 7c, the green group (CG) is roughly clustered on the right side of the OPLS‐DA graph, showing a separation trend from the EHD group in the composition of volatile compounds. CG and EHD treatments have significant differences in the retention of ginger volatile compounds. 13, 17 and 21 kV have a certain aggregation in the left and central regions, indicating that most of the retention of volatile components is similar for the three voltages but there are slight differences between each group. The distinction is not as clear as CG. The three evaluation parameters of the model: *R*_*X*_^2^ and *R*_*Y*_^2^ represent the variance of the *X* and *Y* matrix explained by the model, while *Q* ^2^ represents the predictive ability of the model. These indices close to 1 indicate that the model is more stable and reliable, and a *Q* ^2^ value > 0.5 is considered acceptable for model validity (Zhang, Yan, et al. 2025). The OPLS‐DA of different drying methods, with *R*_*X*_^2^ = 0.991, *R*_*Y*_^2^ = 1, and *Q* ^2^ = 1, indicates that the model quality parameters generated by them all have good stability and predictive ability. Figure 7d shows the results of the OPLS‐DA permutation test, used to evaluate whether the model has a significantly better predictive ability than random classification. After 200 permutation tests, *R* ^2^ = 0.0795 and *Q* ^2^ = −0.774 excluded the possibility of overfitting (Li et al. 2025). The obtained results are of great significance for the identification and analysis of volatile organic compounds in ginger by EHD.

### 3.7 SwissADME Prediction of ADME Properties of Differentiated Compounds

The 27 representative volatile compounds of ginger with a VIP value greater than 1 were selected for SwissADME prediction, and the prediction results are shown in Table 6. Six compounds that met at least four of the drugability rules (i.e., Lipinski, Ghose, Veber, Egan, and Muegge, with at least 4 being ‘Yes’) and had high intestinal absorption (GI) were screened out to identify potential medicinal components. Based on Table 6, compounds such as 6‐Octen‐1‐ol, 3,7‐dimethyl‐, acetate, Bicyclo[2.2.1]heptan‐2‐ol, 1,7,7‐trimethyl‐, acetate, (1S‐endo), 2‐Undecanone, .alpha.‐Bisabolol, Bornyl acetate, and 2‐Undecanol demonstrated certain advantages in drugability. They met multiple drugability rules, indicating that they may have better absorption, distribution, metabolism, and excretion properties in drug development, and their high intestinal absorption also increased the possibility of their exerting effects in the body. These compounds can be used as candidate objects for further pharmacological activity research and provide new directions for drug development. The contents of Bicyclo[2.2.1]heptan‐2‐ol; 1,7,7‐trimethyl‐, acetate, (1S‐endo); 2‐Undecanol in EHD were all higher than those in CG, indicating that EHD retains the medicinal value of ginger well.

**TABLE 6:** Table of predictive rules for druglikeness (Here, “Y” stands for yes, “N” stands for no, “H” stands for high, and “L” stands for low).

Name	CAS	Lipinski	Ghose	Veber	Egan	Muegge	GI
Bicyclo[2.2.1]heptan‐2‐ol, 1,7,7‐trimethyl‐, (1S‐endo)—	464‐45‐9	Y	N	Y	Y	N	H
1,3‐Cyclohexadiene, 5‐(1,5‐dimethyl‐4‐hexenyl)‐2‐methyl‐, [S‐(R*,S*)]—	495‐60‐3	Y	Y	Y	Y	N	L
Camphene	79‐92‐5	Y	N	Y	Y	N	L
Cyclohexane, 1‐ethenyl‐1‐methyl‐2,4‐bis (1‐methylethenyl)‐, [1S ‐(1.alpha.,2.beta.,4.beta.)]—	515‐13‐9	Y	Y	Y	Y	N	L
.beta.‐Myrcene	123‐35‐3	Y	N	Y	Y	N	L
Copaene	3856‐25‐5	Y	Y	Y	Y	N	L
.alpha.‐Phellandrene	99‐83‐2	Y	N	Y	Y	N	L
1,2,4‐Metheno‐1H‐indene, octahydro‐1,7a‐dimethyl‐5‐(1‐methylethyl)‐, [1S‐(1.alpha.,2.alpha.,3a.beta.,4.alpha.,5.alpha.,7a.beta.,8S*)]—	22469‐52‐9	Y	Y	Y	Y	N	L
Aromandendrene	489‐39‐4	Y	Y	Y	Y	N	L
Tricyclo [2.2.1.0 (2, 6)] heptane, 1, 7, 7‐trimethyl—	508‐32‐7	Y	N	Y	Y	N	L
Citronellal	106‐23‐0	Y	N	Y	Y	N	H
Bicyclo[3.1.1]hept‐2‐ene‐2‐carboxaldehyde, 6,6‐dimethyl—	564‐94‐3	Y	N	Y	Y	N	H
6‐Octen‐1‐ol, 3,7‐dimethyl‐, acetate	150‐84‐5	Y	Y	Y	Y	N	H
Bicyclo[2.2.1]heptan‐2‐ol, 1,7,7‐trimethyl‐, acetate, (1S‐endo)—	5655‐61‐8	Y	Y	Y	Y	N	H
2‐Undecanone	112‐12‐9	Y	Y	Y	Y	N	H
.gamma.‐Terpinene	99‐85‐4	Y	N	Y	Y	N	L
Isoborneol	124‐76‐5	Y	N	Y	Y	N	H
endo‐Borneol	507‐70‐0	Y	N	Y	Y	N	H
.alpha.‐Bisabolol	515‐69‐5	Y	Y	Y	Y	N	H
Hexanal	66‐25‐1	Y	N	Y	Y	N	H
Decanal	112‐31‐2	Y	N	Y	Y	N	H
Acetone	67‐64‐1	Y	N	Y	Y	N	H
Bornyl acetate	76‐49‐3	Y	Y	Y	Y	N	H
.beta.‐Bisabolene	495‐61‐4	Y	Y	Y	Y	N	L
Benzene, 1‐(1,5‐dimethyl‐4‐hexenyl)‐4‐methyl—	644‐30‐4	Y	Y	Y	Y	N	L
Naphthalene, 1,2,3,5,6,8a‐hexahydro‐4,7‐dimethyl‐1‐(1‐methylethyl)‐, (1S‐cis)—	483‐76‐1	Y	Y	Y	Y	N	L
2‐Undecanol	1653‐30‐1	Y	Y	Y	Y	N	H

## 4 Discussion

**FIGURE 8:**
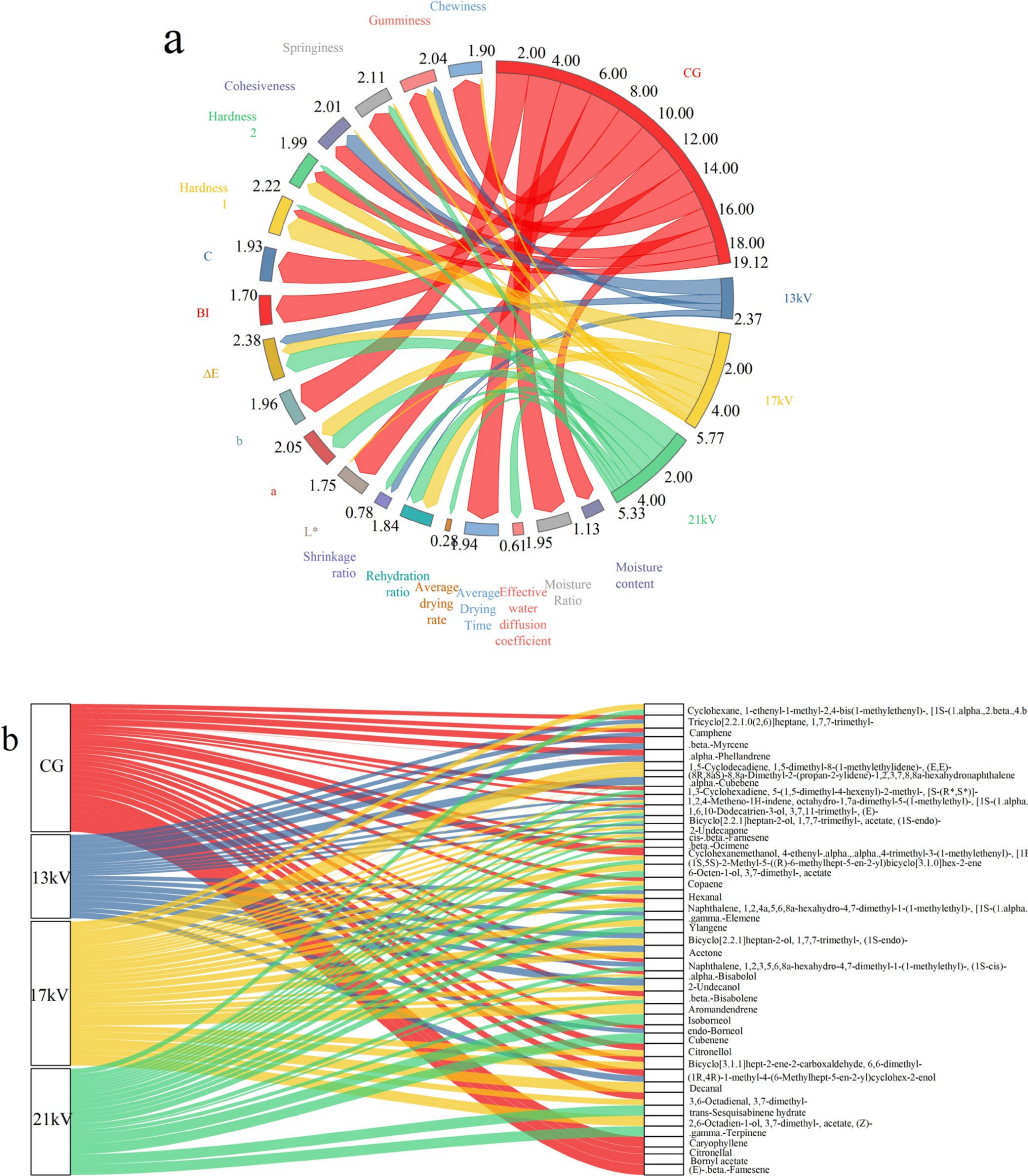
Statistical analysis. (a) Correlation network diagrams of drying characteristics with color and texture parameters; (b) Screening diagram for differential volatile compounds.

Based on the above analysis, the dataset of drying characteristics and color‐textural property data was normalized using z‐score and then a correlation network was plotted as shown in Figure 8a. The differences in multiple detection indicators among the four treatment conditions (CG, 13, 17, 21 kV) were compared. As can be seen from the figure, in terms of drying characteristics, EHD effectively accelerated the drying rate of ginger and reduced the moisture content. There was a significant difference between CG and EHD. In terms of color retention, texture analysis, and rehydration ratio, there were excessive or insufficient phenomena in the CG group. EHD was more in line with the standards of high‐quality ginger than CG, and among the four treatment groups, the 17 kV voltage showed the best performance in terms of color and texture. This indicates that choosing the appropriate voltage is crucial for the drying quality of ginger. Among the ten mathematical models, Midilli et al. had the best prediction effect on the relationship between moisture content and time for ginger. The other models had poor stability across conditions and could not be applied to more complex multi‐variable drying environments.

Figure 8b shows the accumulation diagram of differential volatile compounds in ginger under different drying voltages. The 46 volatile compounds with VIP values greater than 1 were clustered for analysis. The volatile compounds of dried ginger were the most abundant under a voltage of 21 kV, and the retention rate of terpene compounds was the highest, providing the best retention effect for the flavor of ginger. In the SwissADME prediction of the medicinal value of compounds, 6‐Octen‐1‐ol, 3,7‐dimethyl‐, acetate, Bicyclo[2.2.1]heptan‐2‐ol, 1,7,7‐trimethyl‐, acetate, (1S‐endo)‐, 2‐Undecanone, .alpha.‐Bisabolol, Bornyl acetate, 2‐Undecanol showed certain advantages in terms of drugability. Among the four treatment groups, Bicyclo[2.2.1]heptan‐2‐ol, 1,7,7‐trimethyl‐, acetate, (1S‐endo‐) had the highest content under a voltage of 21 kV, and the positive correlation index of 2‐Undecanol at 17 kV was as high as 1.43. Thus, EHD has a high retention rate of volatile compounds with high medicinal value in ginger.

## 5 Conclusions

This study demonstrated, EHD can significantly increase the drying rate and water migration efficiency of ginger, and shorten the drying time. Compared with the control group, the effective water diffusion coefficient under EHD conditions increases, the free water decreases and the bound water ratio increases. In terms of color, the 17 kV group has the best color retention effect, with L* and b* reaching higher levels, a* decreasing, and the chromaticity and hue angle approaching ideal values. In terms of texture, the control group has excessively high hardness, elasticity and chewiness, while the corresponding indicators of the 17–21 kV group decrease. The 21 kV group has a more moderate chewiness and elasticity, which is more acceptable to most people, and the adhesion change is not significant. The LF‐NMR results are consistent with the water distribution analysis, with the free water peak significantly decreasing and the proportion of bound water and restricted water increasing. EHD significantly increases the total amount of volatile organic compounds in ginger, and the retention of terpenoids is significantly improved. Under the 21 kV condition, it is the most conducive to maintaining the aroma characteristics of ginger. Different treatment groups show significant differences in the VOC spectrum. In terms of the mathematical model, Midilli et al. proposed the best fit, with *R* ^2^ approaching 1, *RMSE* and *RSS* were relatively small, and most parameters increase with the increase in voltage. Under the 17 kV condition, it shows the best overall performance, indicating that EHD drying is not necessarily better with higher voltage, and there is an optimal voltage gradient. In conclusion, EHD provides an efficient and low heat damage drying scheme for ginger. It also provides new ideas for further upgrading the drying of heat‐sensitive materials and Chinese medicinal materials by EHD drying technology.

## Author Contributions

**Mingyu Ding:** data curation, software, formal analysis, methodology, validation, visualization, writing – original draft, project administration. **Jingli Lu:** conceptualization, investigation, funding acquisition, writing – review and editing, supervision, resources.

## Funding

The authors are grateful for the support provided by National Natural Science Foundations of China (Nos. 52567018, 12365023, 52067017 and 51467015), Key Project of Innovation and Entrepreneurship for College Students of Inner Mongolia University of Technology (2025096013).

## Consent

The authors have nothing to report.

## Conflicts of Interest

The authors declare no conflicts of interest.

## Data Availability

The data presented in this study are available upon request from the corresponding authors.
